# Why and How the Eyes of Children in the Public Schools Should Be Examined1President’s address before the Medical Society of the County of Erie, January 10, 1899.

**Published:** 1899-02

**Authors:** Lucien Howe

**Affiliations:** Buffalo, N. Y.


### WHY AND HOW THE EYES OF CHILDREN IN THE PUBLIC SCHOOLS SHOULD BE EXAMINED.[1]

BY LUCIEN HOWE, M. D., Buffalo, N. Y.

THIS society has had a corporate existence now for over three quarters of a century, and while the by-law requires the retiring president to present what is called an address, the unwritten law of custom during that long time has required him to deal with some

1. President's address before the Medical Society of the County of Erie, January 10, 1899.

question of general medical interest, preferably some phase of the public health.

I feel under special obligation to discharge also this part of the duty for, as I am informed, this is the first time in the history of the society that any member has held the presidency twice. It is an honor undeserved I fear, but deeply appreciated I know, and one which should be thus properly acknowledged with hearty thanks.

So much has been written on the effect of school life on eye-sight that I would not venture to call attention to so hackneyed a subject were it not that in this vicinity it appears comparatively unknown to the profession in general and to teachers in particular. It is now over a quarter of a century since the first wave of investigation in this direction swept over the world, starting from Europe and advancing thence to America. About thirty years ago and more, when the continent of Europe was mobilising into one vast camp, the conscripting officers found that large numbers of young men were escaping military duty because they were too shortsighted to see the target at the required distance. More systematic investigations were then undertaken to ascertain the causes, and it required but a short time to find that much of the nearsightedness could be traced to school life. These investigations were pushed then in Germany, France, England and elsewhere and the subject was taken up in this country by Agnew and others. In 1876 the Buffalo Medical Association appointed a committee to investigate the effects of school life on the eyes of the children in this city. It happened that I was the chairman of that committee. An examination at that time was made of a little over 1,000 pupils, or exactly 1,003.

The results showed that about the same condition prevailed in Buffalo as had been found elsewhere, the same causes here—defective school furniture and bad light—producing its quota of imperfect vision at the time and the promise of serious disturbances in the future. It was found, for example, that in the primary classes the percentage was exceedingly small of those nearsighted, or who had eyes otherwise imperfect, but that this percentage increased in almost constant ratio with the advancing school years, so that by the time these young men and women were graduated from the High School, 19.7 per cent. were nearsighted or had eyes otherwise defective. In other words, it was impossible to have five children finish the prescribed course without sending one of them out with imperfect vision. Although that was over twenty years ago, there is every reason to believe that the imperfect conditions are practically the same at present.

I would not be understood as finding fault unnecessarily with our public school system. There are plenty to do that, but it is proper to state what the dangers of school-life to vision are, even though a part of these be to a certain extent inevitable. In stating these briefly we must place first of all the production of nearsightedness. It is well known, of course, that the nearsighted or myopic eye is one elongated from before backward, egg-shaped or elipsoidal. We know also that it is produced, in the majority of cases, by hydrostatic pressure. As the person bends forward, the blood is pumped through the arteries into the eye, and if the return circulation is retarded by the force of gravity, by the head being bent over the desk, or secondarily by compression of veins at the neck, then this increased amount of blood in the eye gradually distends the globe.

In a slight degree, of course, nearsightedness is simply an inconvenience easily corrected by glasses, but in the progressive form, or the variety known as malignant myopia, it forms one of the most difficult and dangerous maladies which we are called to treat. But if stooping causes nearsightedness, we naturally ask why the pupil does this and how it can be avoided?

We find the reason for his stooping is:

(*a*) An ill-adjusted desk and seat. The desk must be at a suitable distance from the seat, and the seat of a certain height, preferably the same as the height of the knee. In this way the pupil is enabled to sit upright.

(*b*) The light may be imperfect, requiring the pupil to approach his head to the book, or

(*c*) The light may come from the wrong direction—from the right side, causing the hand to shade what is being written.

A second danger from school-life to the eyes is that if any imperfection in vision already exists, this tendency is increased by the strain which the present school routine demands. Thus, if there exist a tendency to astigmatism, to amblyopia from strabismus, or to any similar disease, this is apt to be aggravated if it passes without correction in early life.

Third.—Contagious diseases of the eyes are frequently propagated in schools—very rapidly, of course, in those school houses where, with utter disregard of hygiene, roller towels are still allowed, and also from the contact which children have with each other in their games there or when coming and returning from school. Under this class of difficulties we have various forms of conjunctivitis, simple catarrhal conjunctivitis and especially granular conjunctivitis.

There are other diseases of the eye which might be mentioned in this connection, but it is possible here only to mention briefly what is well established and in regard to which doctors do not disagree—at least not those who have considered the question at all.

These three classes of diseases of the eye alone are sufficient to indicate why examinations of the eyes of pupils should be made at frequent intervals, at least as often as once a year.

As this paper is restricted to the reasons for these examinations and the proper method for conducting them, it is out of place to consider here any method of treatment. But it goes without saying that such examinations force upon teachers and school boards, and especially upon parents, a sense of the necessity of suitable school furniture, good light, good print and such other precautions as are desirable not only for defective children, but for all pupils as well.

Before leaving this phase of the subject, let me call your attention to certain data bearing on these points presented in the report made last May by the Buffalo School Association on the sanitary condition of our public school buildings. Take first the causes of nearsightedness. First, in regard to school furniture, that report makes the following statement:

"Adjustable desks are entirely wanting in nearly all of the schools. The desks that are used vary little in size, as a rule, in any one room. But in the lower grades of many schools there are large numbers of boys and girls of foreign parentage, just learning the English language, who are far behind their proper grade; these are very often compelled to sit in seats intended for children of half their age. As a consequence, they are not only tortured for the time being, but they are exposed to permanent injury. A very great improvement in this direction could be easily effected by placing more large desks in primary rooms and shifting them about from year to year according to need.

Adjustable desks—now beginning to be much used in other cities—would be a far better remedy still, and would cost little more.

The need of adjustable seats is as noticeable in the new schools as in the old—a fact that will serve as well as any other to point a criticism which the new school buildings deserve—that they show no adequate degree of improvement over the old."

Second, insufficient light:

" There are reported five (5) rooms in which the light is insufficient even on bright days and over one hundred (100) in which the light is insufficient on cloudy days. In forty (40) of this latter number, artificial light is used on cloudy days from a few minutes to all day, depending upon the season of the year and the weather.

Three annexes are overshadowed by the adjoining main buildings, and the light in one of these is so poor at times that work must be stopped.

In one school kerosene lamps are used in two basement rooms."

Third, as to the direction from which the light comes, the following facts are given:

" In quite a number of rooms the light falls on the desks from the wrong direction, that is, from the right side. In at least thirty-five rooms the children face the light to a greater or less extent."

How little the import of such a state of affairs is appreciated is shown by the following instance given in this report:

" The desks in one school room (No. 40) were placed facing the light, because they 'looked better so.' "

In regard to the danger of infection also the report gives valuable testimony. Of the use of towels it says:

" There is no rule as to the use of towels in the schools. Many schools allow none whatever for the children; others have the habit of furnishing a few; for example, one school has one towel every two days for 110 children; another, one a week for 200 children; another, two a week for 138 children."

This report, it will be remembered, was made last May, and it is but fair to assume that the state of affairs which it described is still substantially true. For it should be added concerning all these points that the Superintendent of Education, though keenly alive to these defects, cannot act in that capacity and as medical examiner of schools also. Moreover, most of the defects mentioned come not under his jurisdiction, but under that of the Board of Public Works. None the less, however, they show the reasons why such examinations should be made in Buffalo. Especially do they show that we should have connected with the Board of Health a special officer as examiner of schools. An attempt has already been made to obtain an appropriation for such a medical officer, but thus far without avail.

So much for the reasons why the examinations should be made. Passing next to the second portion, as to how that is done, I

would say first of all that the testing should be made by some teacher rather than by a physician. It is true much greater exactness could be obtained by a physician, particularly if he were an expert at this, but on the other hand, such a plan subjects the examinations to the criticism of officious interference, or to the suspicion that unnecessary prominence is given to the procedure, unless the examiner be a paid member of the health department, and for the ordinary cases this is unnecessary.

Then as to the method of examination, the simpler that it can be made the better. It should be so arranged as to demand of the teacher the least possible knowledge of eyes, be so rapid as to occupy the least possible time of teacher and pupil, and then, in cases where the vision is found to be so imperfect as to necessitate a notice to the parents, this notice should be given in such a manner as to cause as little anxiety as possible. Fortunately our state board of health, realising the necessity of these examinations for pupils in the public schools, has issued a list of instructions to teachers.

This is stated so simply and definitely by Dr. Peter Callan, of New York, a man long identified with ocular hygiene, that it would be unnecessary or confusing to suggest here any modifications. It tells the teacher how to place the pupil at the required distance of twenty feet from the Snellen test types, and then how to write the vision found in the usual fractional form, making distance the numerator and the number of the type the denominator. Having done this, the teacher records on a blank form, recommended also by the state board of health, the name of the pupil, his age, and the vision of each eye. Such a record of the vision, properly made at a certain time, may alone prove of great value to the pupil for future comparison, especially if there be later any tendency to failure of vision. If slight imperfections be discovered, the teacher will naturally take extra precautions as to the position of the child when studying, as to the amount and direction of light and the like. But if the vision is notably imperfect—if it fall below $\frac{20}{40}$ or $\frac{20}{50}$ of normal, then the teacher will prefer—as he should—to place the responsibility where it belongs by notifying the parent in writing of the child's defect. This is rather a delicate matter and should be done with tact and caution. Here again the state board of health has very properly furnished the formula for a printed blank, to be filled out by the teacher and sent to the parent. It reads as follows:

BLANK FORM B.

PUBLIC SCHOOL No.....

MR....................................... .......

The examination of your (son's) daughter's (ward's) eyes, as required by the State Board of Health, shows them to be defective and not up to the standard necessary for satisfactory performance of school work.

You should advise with your family physician as to the choice of an eye doctor, whom you are to consult in the matter.

Respectfully,

..................... ..................Principal.

When parents receive such a printed notice, naturally they turn first to their family physician. If he finds the difficulty is one which he can correct, of course he does so. If not, he refers the patient to some one whom he considers competent. If, however, the circumstances of the parents make it necessary, the family physician refers the parents to some reputable institution where such cases can be seen free of cost. In any event, the child has the attention which he requires, and if the defect proves to be the result of granular lids or some other contagious disease, the examination will have proved an advantage not only to that pupil but to others also.

I have presented the foregoing facts in regard to these examinations of the eyes of children of the public schools partly because of their own importance and partly also because the time is ripe for some definite action to be taken in this direction. I have already conferred with the superintendent of education in regard to the subject, and he is quite in accord with such a movement. The novelty of it, however, to the public, and the fact that it involves even a trifling expense for printing of necessary blanks, tends to a delay which would be less if he were supported by strong medical opinion here. Also the advisability of having a special medical examiner of the schools has been advocated by our health commissioner, but supposed economy has prevented such an addition to the staff of his department. It is probable, however, that if his request were seconded by any considerable number of physicians, it would be granted by the boards of councilmen and aldermen.

As it is customary in addresses of this kind to summarise the facts presented, if possible, in the form of practical suggestions, I would therefore venture the following recommendations :

1. That the attention of the superintendents of education in Buffalo and the towns of this county be called to the advisability of examining the eyes of pupils in the public schools as often as once a year, and that this be done by the teachers in the manner indicated by the state board of health.

2. That this society express to the Mayor, to the boards of councilmen and of aldermen our sense of the necessity of adding to the board of health another physician, who shall not engage in private practice, but who shall give his entire attention to the sanitary condition of the schools and to preserving the health and eye-sight of our seventy odd thousand pupils.

If these two recommendations could be adopted, it is certain that the next generation would suffer less and have better vision than the present one. That improvement in public health, like many others, small by itself, but great in the aggregate, would be only a part of the work inaugurated or guided to completion by this, our medical society of the county of Erie.

183 DELAWARE AVENUE.

